# Retinol-Loaded Poly(vinyl alcohol)-Based Hydrogels as Suitable Biomaterials with Antimicrobial Properties for the Proliferation of Mesenchymal Stem Cells

**DOI:** 10.3390/ijms232415623

**Published:** 2022-12-09

**Authors:** Jeevithan Elango, Camilo Zamora-Ledezma, Daniela Negrete-Bolagay, Piedad N. De Aza, Vicente M. Gómez-López, Ivan López-González, Ana Belén Hernández, José Eduardo Maté Sánchez De Val, Wenhui Wu

**Affiliations:** 1 Department of Biomaterials Engineering, Faculty of Health Sciences, UCAM-Universidad Católica San Antonio de Murcia, Campus de los Jerónimos 135, Guadalupe, 30107 Murcia, Spain; 2 Center of Molecular Medicine and Diagnostics (COMManD), Department of Biochemistry, Saveetha Dental College and Hospitals, Saveetha Institute of Medical and Technical Sciences, Saveetha University, Chennai 600077, India; 3 Green and Innovative Technologies for Food, Environment and Bioengineering Research Group (FEnBeT), Faculty of Pharmacy and Nutrition, UCAM-Universidad Católica San Antonio de Murcia, Campus de los Jerónimos 135, Guadalupe, 30107 Murcia, Spain; 4 School of Biological Sciences and Engineering, Yachay Tech University, Urcuquí 100119, Ecuador; 5 Instituto de Bioingeniería, Universidad Miguel Hernández, Avda. de la Universidad s/n, 03202 Elche, Spain; 6 Tissue Regeneration and Repair Group, Biomaterials and Tissue Engineering, Faculty of Health Sciences, UCAM-Universidad Católica San Antonio de Murcia, Campus de los Jerónimos 135, Guadalupe, 30107 Murcia, Spain; 7 Department of Marine Bio-Pharmacology, College of Food Science and Technology, Shanghai Ocean University, Shanghai 201306, China

**Keywords:** polyvinyl alcohol, retinol, hydrogel, antimicrobial, MSC proliferation

## Abstract

Polyvinyl alcohol (PVA) hydrogels are well-known biomimetic 3D systems for mammalian cell cultures to mimic native tissues. Recently, several biomolecules were intended for use in PVA hydrogels to improve their biological properties. However, retinol, an important biomolecule, has not been combined with a PVA hydrogel for culturing bone marrow mesenchymal stem (BMMS) cells. Thus, for the first time, the effect of retinol on the physicochemical, antimicrobial, and cell proliferative properties of a PVA hydrogel was investigated. The ability of protein (3.15 nm) and mineral adsorption (4.8 mg/mL) of a PVA hydrogel was improved by 0.5 wt.% retinol. The antimicrobial effect of hydrogel was more significant in S. aureus (39.3 mm) than in E. coli (14.6 mm), and the effect was improved by increasing the retinol concentration. The BMMS cell proliferation was more upregulated in retinol-loaded PVA hydrogel than in the control at 7 days. We demonstrate that the respective in vitro degradation rate of retinol-loaded PVA hydrogels (RPH) (75–78% degradation) may promote both antibacterial and cellular proliferation. Interestingly, the incorporation of retinol did not affect the cell-loading capacity of PVA hydrogel. Accordingly, the fabricated PVA retinol hydrogel proved its compatibility in a stem cell culture and could be a potential biomaterial for tissue regeneration.

## 1. Introduction

Polyvinyl alcohol (PVA) is a promising biodegradable, hydrophilic, and biocompatible synthetic polymer that has been extensively involved in different sectors of the biomedical field [1]. Due to its unique features, PVA has been used in recent times in tissue engineering applications for artificial organ development [2,3]. PVA-based hydrogels are a colloidal dispersion with three-dimensional network structures. This material has great potential due to its low toxicity, high water absorption, good mechanical properties (elastic modulus and mechanical strength), biocompatibility, and good physicochemical properties [4,5]. For instance, PVA hydrogel was potentially useful in cartilage tissue substitutes [6], heart valves [7], arterial phantoms [8], corneal implants [9], electroskins, actuators, supercapacitors, and fuel cells [10,11]. Due to the flexible crosslinking ability, PVA hydrogel was fabricated with different biomolecules and polymers, such as carboxyl methyl cellulose [12], gelatin, chitosan [13], graphene oxide [14], MXene [11], lignin-silver hybrid nanoparticles [15], agar [16], and laponite [17]. Due to its excellent biocompatibility, PVA hydrogel is widely used in tissue engineering applications [18,19,20]. Moreover, the safety aspects of PVA hydrogel have been tested in different in vitro cell culture systems using MC3T3-E1 (mouse osteoblastic) [21], NIH-3 T3 (mouse embryonic fibroblast), human chondrocytes [22], mesenchymal stem (MSCs) [23], MRC-5 (human fibroblast) [24], and SV40 (human osteoblast) [25]. The antibacterial effect of PVA hydrogels was tested against bacteria (B. subtilis, S. aureus, and E. coli), mold (Rhizopus and Asp. niger and Asp. oryzae), and fungus (C. albicans) [26].

Retinol (vitamin A) is an essential fat-soluble vitamin that plays a major role in normal tissue growth and biological function. In low concentrations, retinol is a powerful antioxidant and free radical scavenger that also helps the synthesis of collagen and elastin, which contributes to skin hydration and radiance. In high concentrations, it has keratolytic activity, reducing the stratum corneum (cell renewal), regenerating wrinkles and acne marks, and depigmenting properties [27]. In addition, several studies have already proven the beneficial effects of retinol on the immune and therapeutic systems [28]. In addition, the cell proliferative effect of retinol was substantially proven in several studies performed with stem cells [29], fibroblasts [30], chondrocytes [31], keratinocytes, and bone cells [32,33]. Recently, the use of retinol in biomedical applications is an increasing trend, and several novel approaches have been initiated to better use and overcome the major drawbacks of retinol in the practical field. One of the major problems of retinol is stability, which tends to degrade faster due to its unstable chemical structure, and it has also been reported that retinol has a narrow therapeutic window, making it ineffective at low concentrations and toxic at high concentrations [27]. Therefore, several approaches have been tried with encapsulating or doping retinol in polymer-based systems, with hydrogel biomaterials being one of the most promising. Indeed, the most suitable functionalized hydrogel for biomedicine applications should exhibit the following characteristics: nontoxic (inertness), biodegradable macromolecules, and self-healing for lifespan extension. Additionally, it is also desired for tissue engineering that such materials intrinsically show improved mechanical properties (such as hardness and elasticity) and structure (such as porosity and density) in order to be compatible with embedded tissues. So far, the development of multifunctional hydrogels to meet complex biological conditions in vivo and achieve the adequate synergy with live organisms remains a current challenge [34]. Based on the concept, among the different polymers used to date, PVA deserves especial attention because it could be an excellent polymer that has several beneficial effects and is easy to form complex microstructures. However, the potential application of retinol-loaded PVA hydrogel in biomedical application has unfortunately never been investigated until now. Considering the significance, for the first time, the present study aimed to fabricate a retinol-loaded PVA hydrogel system and test the mechanical, functional (protein and mineral adsorption), antimicrobial, and MSC proliferative/cell-loading capacity of hydrogels.

## 2. Results

### 2.1. Maximum Absorbance of Hydrogel

**Figure 1:**
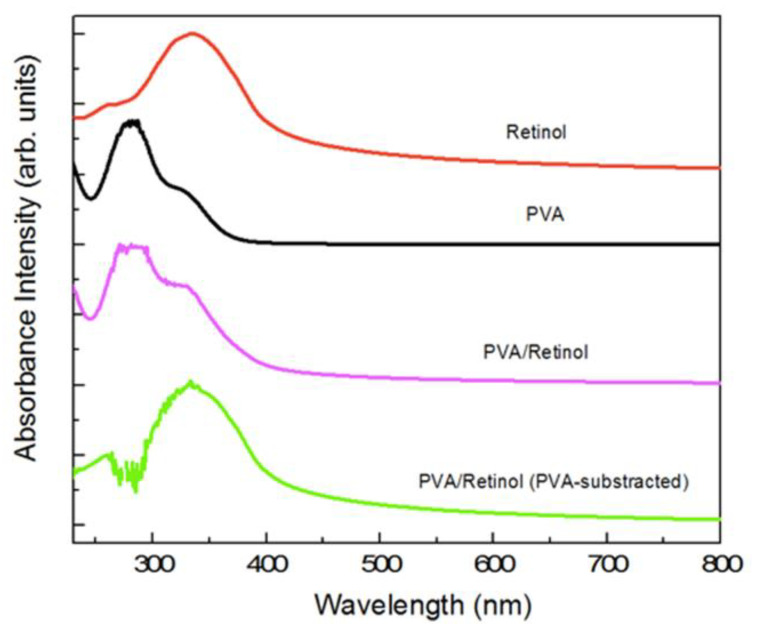
Representative UV-Vis spectra of PVA and retinol-loaded PVA. From top to bottom: i. Retinol aqueous solution containing 0.02 wt.% of retinol (0.2 mg/mL); ii. PVA aqueous solution containing 15 wt.% of PVA (150 mg/mL); iii. PVA/retinol hydrogel containing 150/0.2 mg/mL of PVA and retinol, respectively; and iv. PVA/retinol hydrogel (PVA-substracted) shows the spectrum of PVA/retinol hydrogel containing 150/0.2 mg/mL of PVA and retinol, respectively, by subtracting the PVA as background in which the retinol absorbance peak at 334 nm is clearly observed. All spectra were normalized and shifted along the Y axis for clarity.

The maximum absorbance spectra of an aqueous solution and hydrogels loaded with retinol are shown in Figure 1. It shows the UV maximum absorption of PVA (150 mg/mL) and retinol (0.2 mg/mL) in an aqueous solution. The retinol concentration for these experiments was chosen based on the initial stage maximum absorbance of around 1 at 334 nm. As shown in Figure 1, the typical spectrum for the hybrid PVA/retinol hydrogel composite containing 150/0.2 mg/mL showed both peaks of PVA and retinol; however, subtracting the PVA peak clearly showed the typical absorbance with a main broad peak of retinol at 334 nm, demonstrating the presence of intact retinol in the hydrogel. The peak intensity of PVA was also significantly altered and shifted slightly to a higher wavelength by retinol incorporation.

### 2.2. Retinol Degradation

**Figure 2:**
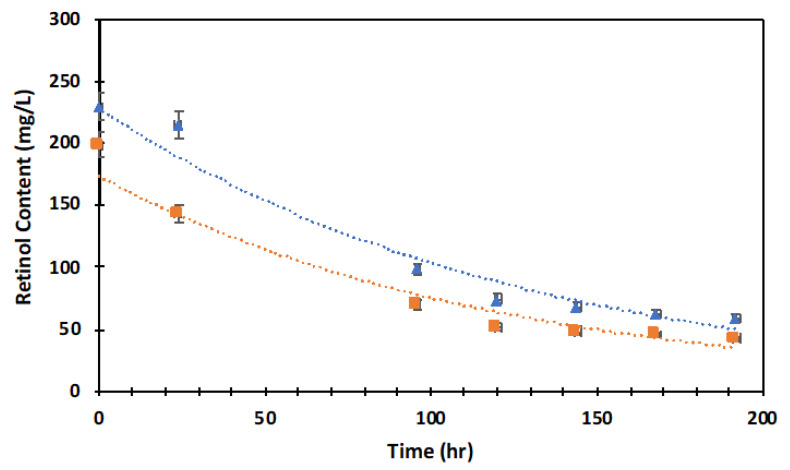
Retinol degradation as a function of the time for an aqueous solution of retinol (200 mg/L) (blue triangles) and a retinol (200 mg/L)-loaded PVA (150 g/L) hydrogel (orange square) incubated at 37 °C. Retinol content was computed through the intensity of the absorbance at different periods. Discontinuous line represents exponential fit as RC = 228 e(−0.008t) and RC = 173 e(−0.008t) for the aqueous suspension and hydrogel, respectively. In both series of samples, retinol was degraded up to between 75 and 78% in 8 days of incubation at 37 °C with a similar decay rate at −0.008. RC = Retinol Content.

The retinol molecule is known to degrade readily due to UV/temperature exposure. In order to guarantee that our samples were suitable as a biomaterial for a cell culture, we evaluated their degradation over time (8 days). Thus, retinol’s degradation both alone in an aqueous solution and crosslinked within hydrogel was investigated by using UV absorption (334 nm) at 37 °C. In both series of samples, retinol was degraded up to between 75 and 78% in 8 days of incubation (Figure 2). It is worth mentioning that the degradation rate of retinol in the aqueous solution or within the hydrogel showed a similar decay rate at −0.008 within the period studied, suggesting that the molecular structure was conserved and degraded similarly in both the solution and hydrogels. It is also worth noting that due to the detection limit of the UV-Vis spectrometer used in these experiments, the retinol content was much lower than the loading used in our set of hydrogel biomaterials (0.1 wt.% < retinol < 0.5 wt.%). Thus, our set of samples was designed to guarantee a controlled retinol delivery in the media during the 8 days of biological assays despite the high degradation rate observed in this system.

### 2.3. Textural Analysis

**Figure 3:**
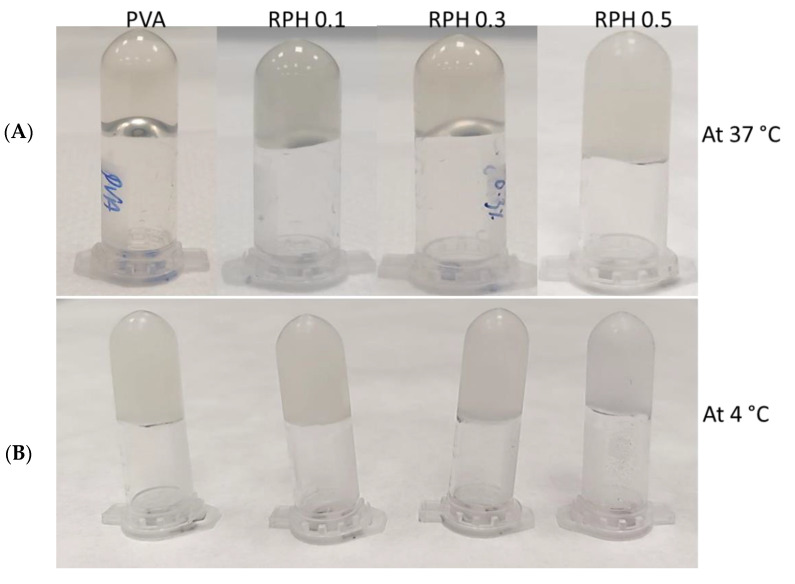
Gelling behavior of retinol-loaded PVA hydrogel at 37 °C (**A**) and 4 °C (**B**). PVA solution 15 wt%, RPH 0.1-0.1% retinol PVA, RPH 0.3-0.3% retinol PVA, and RPH 0.5-0.5% retinol PVA hydrogel.

The textural analysis results demonstrate that neither hardness nor consistency in our retinol-loaded hydrogels showed significant differences if compared with their native (PVA) hydrogel counterparts. Table 1 summarizes the values obtained from the textural analysis. In fact, the gel strength of PVA hydrogel at 4 °C was 0.29 N, and the addition of retinol did not alter the gel strength compared to the control group (Figure 3).

### 2.4. Protein Adhesion Ability

**Figure 4:**
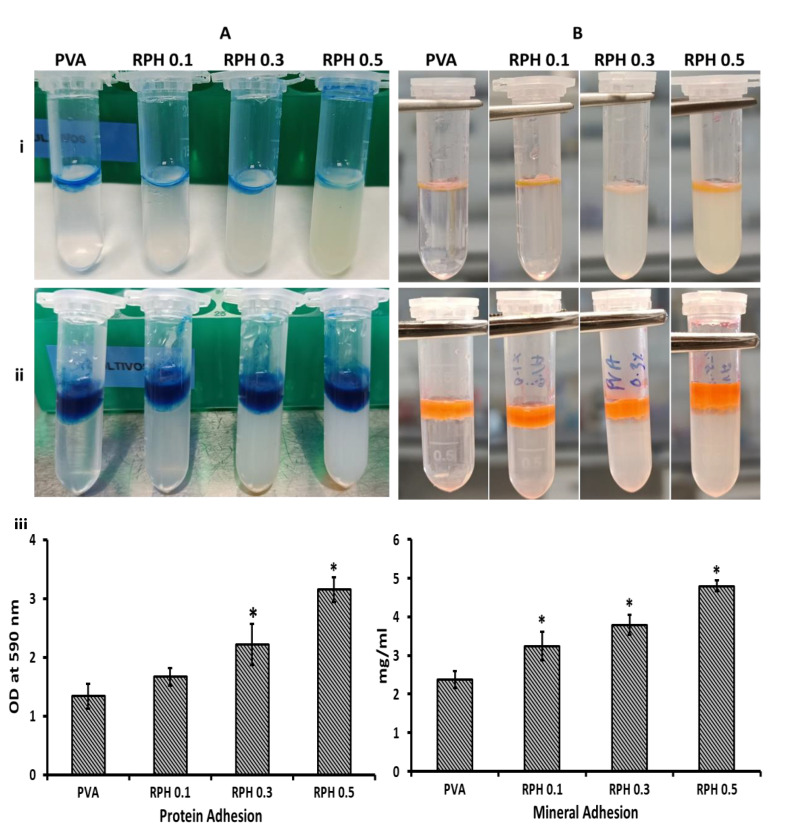
The upper panel shows representative macroscopic images of the RPHX hydrogels stained for protein and mineral adhesion assessments. The lower panel shows Optical Density (OD) measured at 590 nm for both protein (**A**) and mineral adhesion (**B**) assessment in the RPHX hydrogels, respectively. PVA solution 15 wt.%, RPH 0.1-0.1% retinol PVA, RPH 0.3-0.3% retinol PVA, RPH 0.5-0.5% retinol PVA hydrogel. (i) Negative control containing PVA/retinol without FBS or biomimetic mineral treatment, (ii) Test samples with FBS or biomimetic mineral treatment, and (iii) UV absorption of protein and mineral adhesion (No UV absorption was observed in negative control samples). * represents statistical significance, * *p* < 0.05 vs. PVA.

The ability of hydrogel in protein adhesion was investigated by the CBB staining method. The results show that all hydrogels tended to adhere to the protein on their surface (Figure 4A). Interestingly, increasing the retinol concentration increased the protein-binding ability of the hydrogel significantly. The negative control without FBS had no absorbance at the respective wavelength. Compared to the control, retinol-incorporated hydrogel (except 0.1 wt.% of incorporated hydrogel) had a significantly higher protein adhesion (*p* < 0.05). Notably, the higher concentration (0.5 wt.%) of retinol-loaded hydrogel had a twofold higher protein-binding ability compared to the control hydrogel (*p* < 0.05). 

### 2.5. Mineral Adhesion Ability

To understand the mineral-binding ability of hydrogel, the amount of synthesized bone mineral deposition on hydrogel was evaluated by the alizarin red method. It is clearly shown that the range of mineral deposition was improved by retinol in a dose-dependent manner and all the hydrogel had excellent mineral adhesion behavior (Figure 4B). Like protein adhesion, the negative control had no absorbance peak. The mineral-binding ability of hydrogel was significantly improved by 0.3 and 0.5 wt.% of retinol incorporation compared to the control group (*p* < 0.05). Like protein adhesion, the hydrogel with 0.1 wt.% retinol did not contribute a significant change compared to the control. As expected, a higher mineral deposition rate was observed in the higher concentration of retinol (0.5 wt.%)-loaded PVA hydrogel.

### 2.6. Antimicrobial Activity of PVA Retinol Hydrogel

**Figure 5:**
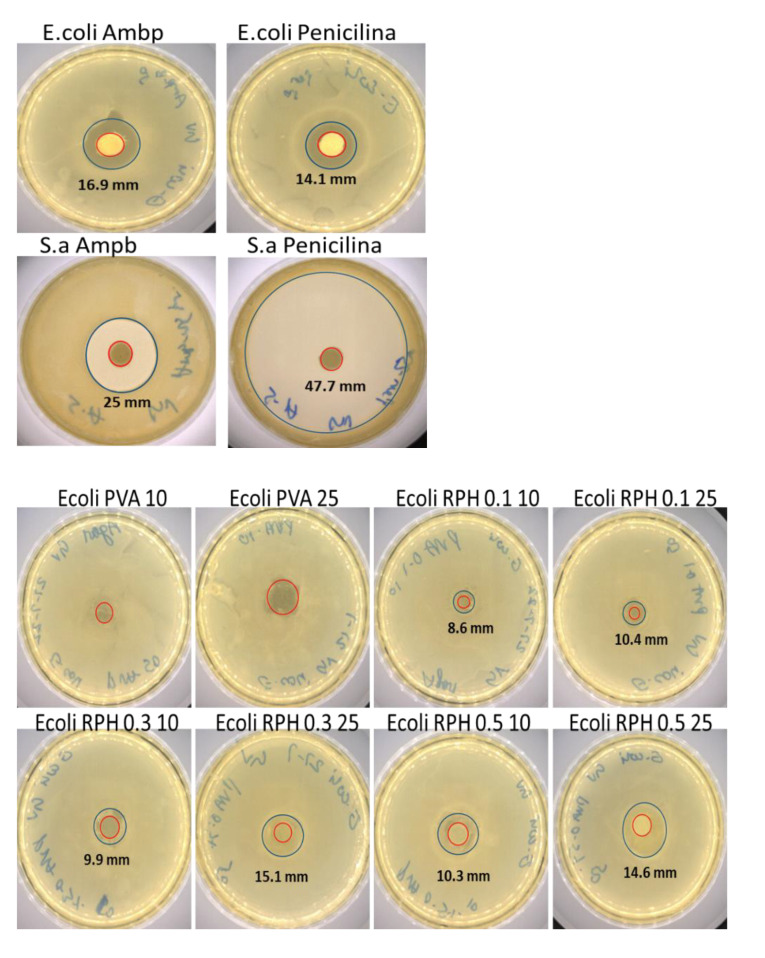

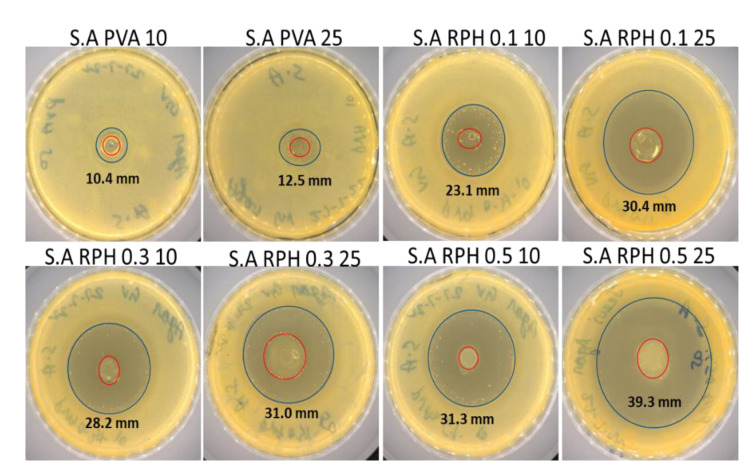
Representative macroscopic images of the antibacterial capacity of the RPHX hydrogels. The upper panel shows positive control of *E. coli* and *S. aureus* using amphotericin B (20 μL) and penicillin (20 μL). The lower panel shows the antibacterial efficacy of RPHX by using the disk diffusion method using both *E. coli* (mm) and *S. aureus* (mm) strains. Control strains treated without hydrogel, PVA solution 15 wt.%, RPH 0.1-0.1% retinol PVA, RPH 0.3-0.3% retinol PVA, RPH 0.5-0.5% retinol PVA hydrogel. The numbers 10 and 25 correspond to strains treated with 10 μL and 25 μL of RPHX hydrogel, respectively.

For the antimicrobial activity of hydrogel, two methods, disk diffusion and microbial adhesion, were performed with two different bacterial strains, *S. aureus* and *E. coli,* and hydrogel concentrations (10 and 25 µL), respectively. The results show that PVA hydrogel with 10 and 25 µL did not show any antimicrobial activity against *E. coli* (Figure 5). The bacterial strains treated with 20 µL of antibiotics (penicillin and amphotericin B) served as the positive control group. Amphotericin B showed a higher inhibition zone (16.9 mm) against *E. coli* than penicillin (14.1 mm); in contrast, the inhibition rate of penicillin was higher (47.7 mm) than amphotericin B (25 mm) against *S. aureus* (Table 2). 

Interestingly, the antimicrobial activity of PVA hydrogel against *E. coli* was improved by the retinol (0.1–0.5 wt.%). For instance, bacterial inhibition was clearly seen with the treatment of hydrogel incorporated with retinol, and the rate of inhibition was about 8.6–14.6 mm with 0.1–0.5 wt.% of retinol (10 and 25 µL), respectively (Table 2). Unlike the antimicrobial effect against *E.coli*, PVA hydrogel showed an inhibitory effect against *S. aureus,* and the inhibition effect (10.4 and 12.5 mm) was more pronounced with the increasing concentration of PVA hydrogel from 10 to 25 µL, respectively. The higher concentration of retinol showed a similar inhibition effect against *E. coli* as compared to the positive control, penicillin; however, it was lower than amphotericin B.

Like the previous result, the inhibitory effect of retinol against *S. aureus* was in a dose-dependent manner. The rate of antimicrobial inhibition was about 23.1–39.3 mm in different concentrations (10 and 25 µL) of retinol (0.1–0.5 wt.%)-loaded hydrogel, and the inhibitory effect (39.3 mm) was higher at 0.5 wt.% of retinol-incorporated PVA hydrogel. Compared to the control hydrogel, all the retinol-incorporated hydrogel had a significantly higher antimicrobial inhibition effect (*p* < 0.05). Surprisingly, the inhibitory effect of 0.5 wt.% of retinol-incorporated PVA hydrogel against *S. aureus* was comparable with the positive control group treated with commercial antibiotics, such as penicillin and amphotericin B (Figure 5).

### 2.7. Microbial Attachment 

**Figure 6:**
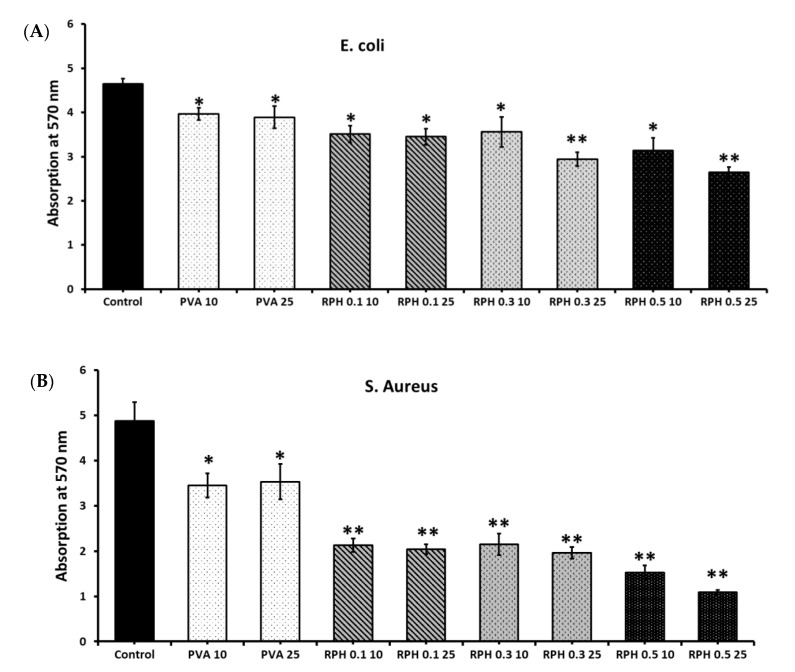
Microbial attachment of RPHX hydrogels with *E. coli* (**A**) and *S. aureus* (**B**) strains. Control strains treated without hydrogel, PVA solution 15 wt.%, RPH 0.1-0.1% retinol PVA, RPH 0.3-0.3% retinol PVA, RPH 0.5-0.5% retinol PVA. The numbers 10 and 25 correspond to strains treated with 10 μL and 25 μL of RPHX hydrogel, respectively. * represents statistical significance, * *p* < 0.05 vs. control, ** *p* < 0.001 vs. control.

The ability of the hydrogel to attach to bacterial cells was evaluated by culturing the respective strains on the hydrogel for 24 h and quantified at 570 nm. The rate of microbial attachment in both *E. coli* and *S. aureus* decreased in the hydrogel-treated group compared to the control group (Figure 6). In addition, increasing the retinol concentration from 0.1 to 0.5 wt.% significantly decreased the rate of microbial attachment of PVA hydrogel to *S. aureus* and *E. coli*. Of the hydrogels cultured in the two microbial groups, the lowest cell attachment was observed in the 25 µL of 0.5 wt.% of retinol PVA hydrogel-treated *S. aureus* group. Interestingly, the rate of microbial cell attachment was significantly lower in the PVA hydrogel and retinol PVA hydrogel with *S. aureus* than in *E. coli*, which substantially supported the zone of inhibition of PVA retinol hydrogel against *S. aureus* and *E. coli* (Figure 5). 

### 2.8. The Effect of BMMS Cells 

**Figure 7:**
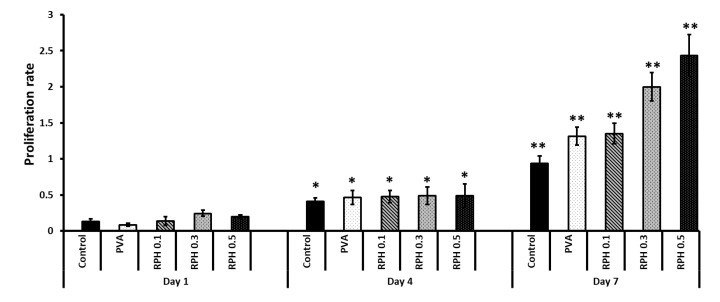
The effect of RPHX hydrogels on the proliferation of bone marrow mesenchymal stem cells cultured for 1, 4, and 7 days. Control cells treated without hydrogel, PVA solution 15 wt.%, RPH 0.1-0.1% retinol PVA, RPH 0.3-0.3% retinol PVA, RPH 0.5-0.5% retinol PVA. Asterisks represent statistical significance, * *p* < 0.05 vs. Day 1, ** *p* < 0.001 vs. Day 1 of respective group.

**Figure 8:**
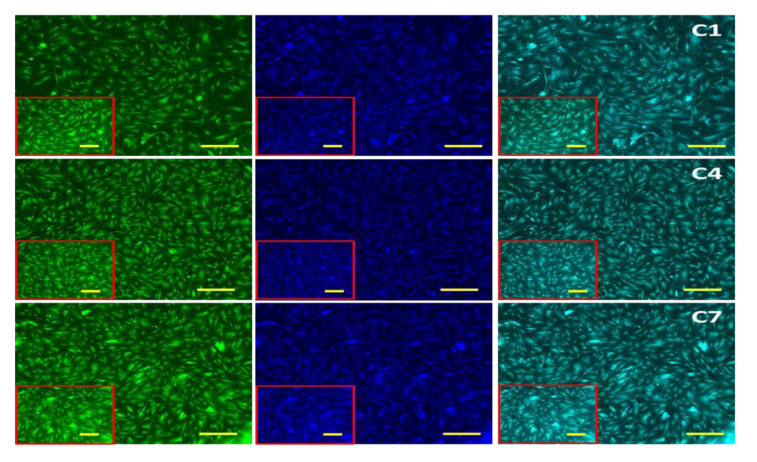

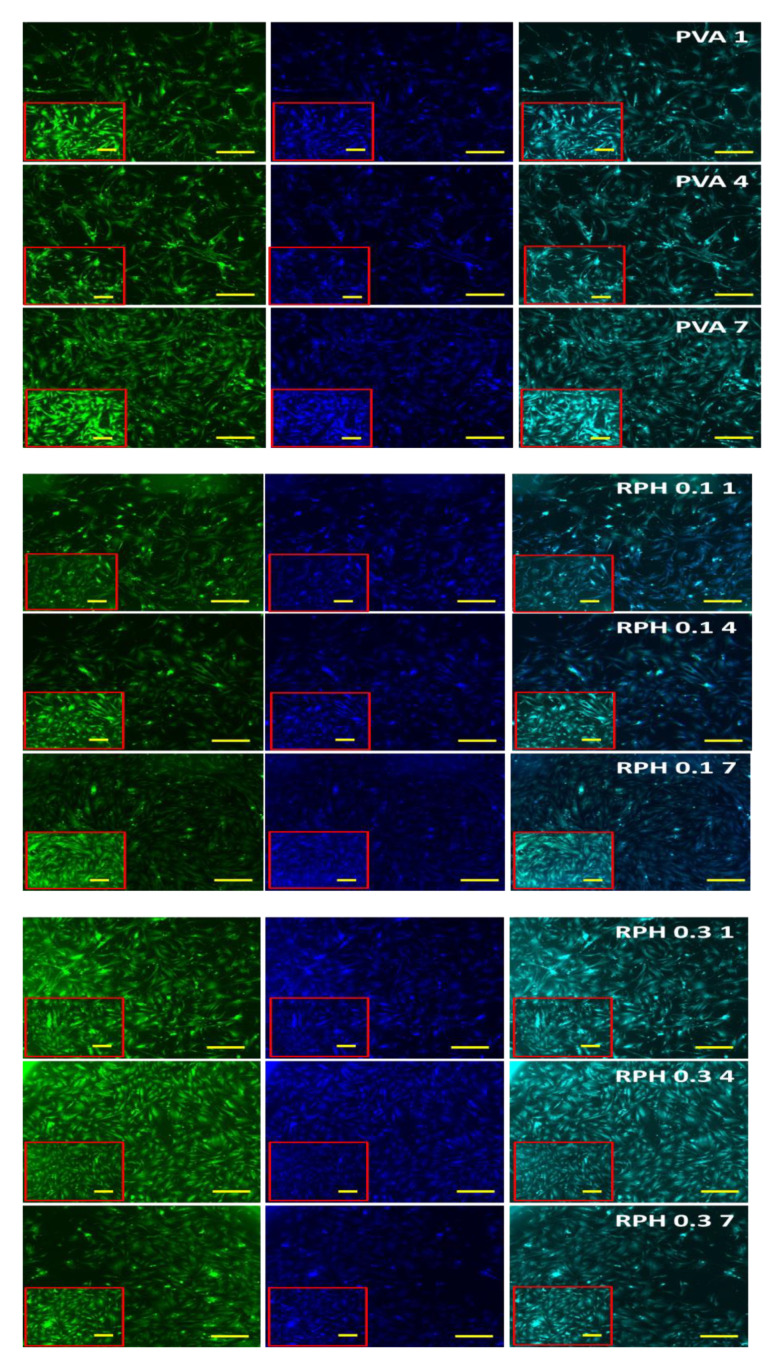

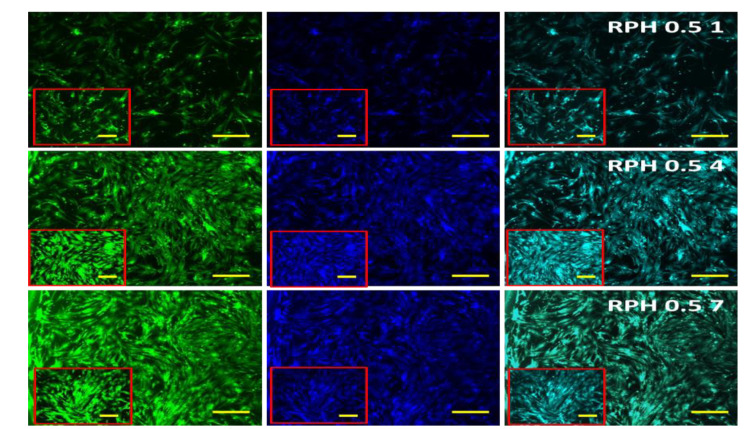
The FITC and DAPI fluorescence images of bone marrow mesenchymal stem cells cultured in RPHX hydrogels for 1, 4, and 7 days. Control cells treated without hydrogel, PVA solution 15 wt.%, RPH 0.1-0.1% retinol PVA, RPH 0.3-0.3% retinol PVA, RPH 0.5-0.5% retinol PVA. Scale bar 5×—200 μm, 10×—100 μm (Insert).

**Figure 9:**
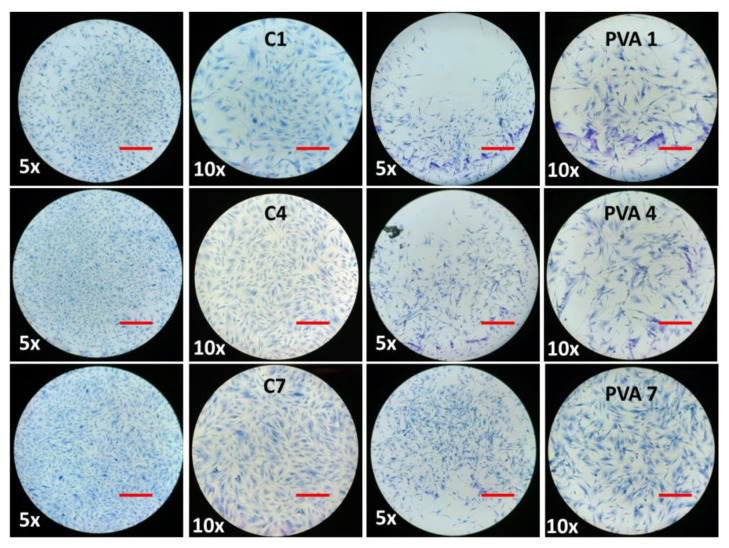

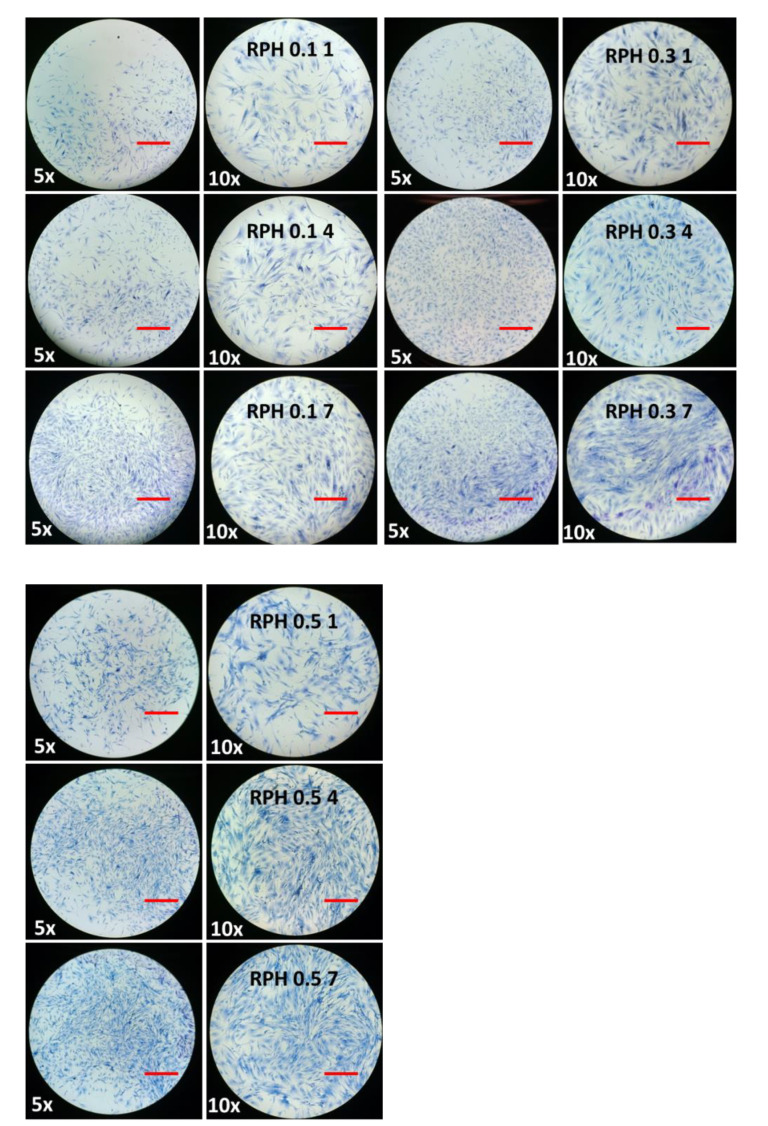
The hematoxylin- and eosin-stained images of bone marrow mesenchymal stem cells cultured in RPHX hydrogels for 1, 4, and 7 days. Control cells treated without hydrogel, PVA solution 15 wt.%, RPH 0.1-0.1% retinol PVA, RPH 0.3-0.3% retinol PVA, RPH 0.5-0.5% retinol PVA. Scale bars 5×—200 μm, 10×—100 μm.

The biocompatibility of hydrogel in BMMS cell growth was determined by culturing BMMS cells with hydrogel for 1, 4, and 7 days and investigating them by the MTT method, H&E staining, and fluorescence microscopy at the respective time points. As shown in Figure 7, the proliferation rate of BMMS cells was significantly upregulated by the hydrogel treatment (*p* < 0.05). However, there was no significant change observed between the control and the BMMS cells treated with hydrogel for 1 and 4 days. In contrast, the cell proliferation rate was accelerated in the PVA and PVA retinol hydrogel-treated groups compared to the control group on day 7. The PVA hydrogel with 0.1 wt.% and 0.5 wt.% of retinol showed a similar effect with the PVA and PVA 0.3 wt.% of retinol hydrogel, respectively. Like the results observed in Figure 7, the FITC and DAPI fluorescence staining intensity of the BMMS cells cultured with PVA retinol hydrogel was higher than the control group, especially on day 7 in PVA 0.5 wt.% of retinol hydrogel (Figure 8), which was further confirmed by the H&E staining images of the BMMS cells (Figure 9). 

### 2.9. BMMS Cell-Loading Capacity of the Hydrogel

**Figure 10:**
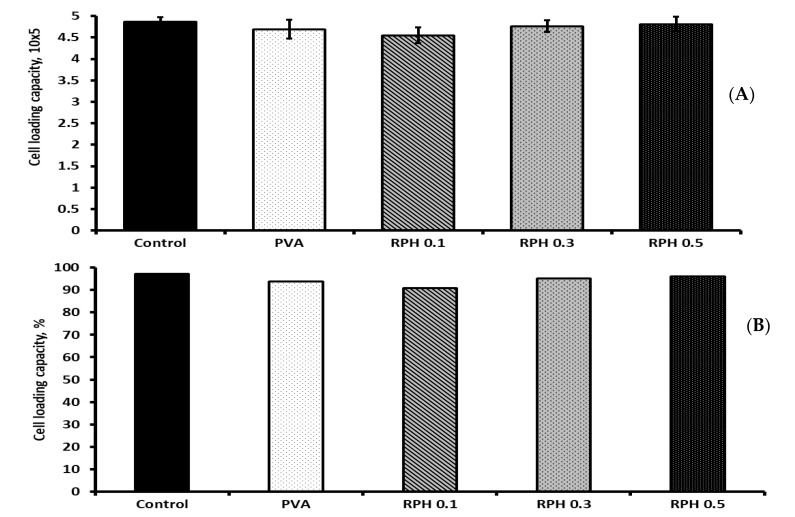
Cell-loading ability actual (**A**) and percentage (**B**) of RPHX hydrogels using bone marrow mesenchymal stem cells for 6 h. Control cells treated without hydrogel, PVA solution 15 wt.%, RPH 0.1-0.1% retinol PVA, RPH 0.3-0.3% retinol PVA, RPH 0.5-0.5% retinol PVA.

To investigate the cell-loading ability of hydrogel, BMMS cells with a cell density of 5 × 10^5^ cells were loaded on hydrogel and cultured for 6 h. At the end of the experiments, the total number of cells in the control, PVA, RPH 0.1, RPH 0.3, and RPH 0.5 was about 4.86, 4.69, 4.55, 4.76, and 4.81 × 10^5^, respectively (Figure 10). The overall cell-loading capacity was 93.8% for PVA hydrogel and 91–96.2% for retinol (0.1–0.5 wt.%)-loaded hydrogels, respectively; however, no significant changes were observed between the PVA and retinol (0.1–0.5 wt.%)-loaded PVA hydrogel groups. In the same way, increasing the retinol concentration from 0.1 to 0.5 wt.% did not produce any significant changes in the BMMS cell-loading capacity of the hydrogel. In addition, the percentage of cell-loading capacity in the control group was 97.2%, which was comparable (96.2%) to the PVA hydrogel with a higher retinol (0.5 wt.%) concentration.

## 3. Discussion

In the present study, the UV absorption peak at 334 nm of PVA/retinol hydrogel confirmed the successful accumulation of retinol in the hydrogel. Like the present study, the maximum absorption peak of retinol was reported at 325 nm [35,36,37]. The shifting peak wavenumber of PVA retinol hydrogel to a higher frequency than PVA hydrogel might be due to the chemical interaction between PVA and the retinol compounds. In an earlier study, the characteristic peak of PVA hydrogel loaded with ZnO was reported at 260 nm [38]. Like the present study, the maximum absorption peak for PVA was observed at 275 nm, and the peak was shifted to a higher wavenumber to 282 and 300 nm by 0.05 g and 0.15 g CuO [39]. In another study, the absorption peak of PVA hydrogel was shifted to 428 nm by chitosan and silver nanoparticles [40]. 

Previously, Failloux et al. investigated the degradation pattern of retinol by using high-performance liquid chromatography (HPLC) with UV/Vis spectroscopy [41]. It has been reported that retinol tends to easily degrade against UV [42], which was stabilized by a lipid nanocarrier [43]. In the present study, the degradation rate of retinol was not altered by PVA hydrogel and showed a similar pattern of degradation between retinol and retinol hydrogel. In the present study, we used the hydrogel containing 150 g/L of PVA and 200 mg/mL of retinol due to the optimal absorbance of the UV-Vis spectrophotometer. Indeed, the higher retinol concentration (200 mg/L) was chosen based on the maximum absorbance (around 1), and we obtained a retinol content = 228 exp(−0.008t) and RC = 173 exp(−0.008t) for the aqueous suspension of retinol and its hydrogel, respectively, according to the exponential fit of the experimental data. In both series of samples, retinol was degraded up to 75–78% at 37 °C with a similar decay rate at −0.008.

It has been reported that the gel strength of PVA was improved by the addition of chitosan through the hydrogen bond interaction [44,45,46,47,48]. The gel strength obtained in the present study did not show significant differences, and the values obtained were comparable with similar polymer-based hydrogels reported in the literature [49]. The gel strength of the PVA/retinol hydrogel was more stable at 4 °C compared to 37 °C. Furthermore, the values obtained were comparable with similar polymer-based hydrogels reported in the literature [50]. In contrast, compared to 4 °C, the PVA hydrogel had a low gel strength at 37 °C, which was below the measurable range by the texture analyzer. It is worth noting that the measurement of the hardness in gel-like structures is commonly related to the strength of the hydrogels and their inner crosslinked structure. Indeed, the measurement of the gel strength in gel-like structures is commonly related to the strength of the hydrogel [50,51]. In the present study, the presence of retinol within the PVA hydrogel matrix did not disturb the intrinsic crosslinking of the hydrogel, but instead, the values were almost constant, which means that the mechanical properties of the gel were not altered by the presence of the retinol molecules at the load content (1 mg/mL < RC < 5 mg/mL). Overall, all these findings show that fabricated hydrogel is highly desirable for the fabrication of tailored hydrogel loaded with bioactive molecules. 

The surface absorption of plasma protein highly correlates with the interaction of hydrogel with the immune system, blood biocompatibility, activation, and adherence of platelets [52,53]. In the present study, retinol significantly favored the protein adsorption behavior of the PVA hydrogel, especially the higher concentration of retinol at 0.5 wt.%. To support the present finding, an earlier report suggested that the protein adsorption ability of PVA/HA/CH was increased up to ~94% compared to the PVA/HA control (65–72%) due to the high porosity and swelling behavior of chitosan [54]. Similarly, Hwang et al. [55] reported that blended hydrophilic biomaterials, such as alginate or dextran, could increase the protein adsorption rate of the PVA/dextran scaffold. The protein adsorption of the PVA hydrogel fabricated with 1-Vinyl-3-butylimidazolium bromide and acrylamide was about 37.25–147.75 mg/g [26]. Based on the previous study, the surface protein absorption of PVA was accelerated by hyaluronic acid-based nanofibers through physical bonds, e.g., hydrogen bonding, ionic bonding, hydrophobic interactions, and Van der Waal interactions [56]. It was also opined that the protein adsorption behavior of the PVA hydrogel depends on the electrostatic interaction, hydrophobicity surface chemical groups, and surface energy between the hydrogel surface and the proteins [57,58,59]. Based on this evidence, the PVA hydrogel surface is significantly altered by the retinol content and thereby facilitates more adsorption of protein on the hydrogel.

To understand the ability of hydrogel in the mineralization process, biomimetic apatite was commercially synthesized in the work and used for the mineral adhesion behavior of hydrogel by using alizarin red staining. The range of mineral deposition was higher in the hydrogel with a high content of retinol (0.5 wt.%) compared to the control. To support this finding, previously Shi et al. fabricated PVA hydrogel with biomimetic mineral hydroxyapatite for cartilage repair [60]. Ye et al. fabricated a PVA scaffold by coating it with biomimetic apatite [61]. Interestingly, all the hydrogels including the control showed excellent mineral deposition behavior, which ultimately proves the suitability of this hydrogel system in bone and extracellular matrix formation. 

The antimicrobial experiment showed that the PVA hydrogel showed more inhibition activity against *S. aureus* compared to *E. coli*. Similarly, previous studies also reported the antimicrobial characteristics of PVA hydrogel fabricated with starch [62], chitosan [63,64], polyvinyl pyrrolidone [65], curcumin [63], zinc oxide nanoparticles [64], gelatin [66], chitooligosaccharides conjugated with gallic acid [67], and silver nanoparticles [68]. For decades, retinol has been potentially applied for several clinical conditions due to its anti-infective effect [69,70,71]. In the present study, the antimicrobial activity of PVA hydrogel was significantly improved by the retinol content in the hydrogel. The antimicrobial activity of retinol against different bacterial strains, including *S. aureus* and *E. coli,* was reported by several authors [71,72,73,74,75], which substantially supports the present result. Earlier empirical evidence claimed that the antimicrobial property of retinols to protect skin and the antimicrobial effect of retinol are due to the stimulation of immunity-related gene expressions responsible for retinoic acid response elements [72]. More specifically, Wheelwright et al. reported the actual mechanism whereby the antimicrobial activity of vitamin A against M. tuberculosis was regulated by NPC2-dependent expression and function [76]. The IC50 value of retinol was reported to be 4–8 mg/mL for methicillin-sensitive and methicillin-resistant *S. aureus*, respectively [71].

Notably, the proliferative effect was significantly improved in BMMS cells cultured with PVA hydrogels compared to the control, which substantially proved the efficiency of PVA hydrogel in the MSC cell culture. To support this finding, earlier studies disclosed the proliferative effect of MSC cells cultured in PVA hydrogel alone [23,77,78] or fabricated with chitosan [79,80], chitosan methacrylate [81], hyaluronic acid [82], poly(2-methacyloyloxyethyl phosphorylcholine (MPC)-co-n-butyl methacrylate (BMA)-co-p-vinylphenylboronic acid (VPBA)) [83], silk fibroin [84], and heparin [85].

The BMMS cells’ proliferative effect of PVA hydrogel was significantly improved by the incorporation of retinol from 0.1 to 0.5 wt.%. Several studies already claimed the beneficial effect of retinol in stem cell proliferation, metabolism, and differentiation [86,87,88,89,90,91]. The differentiation potential of mesenchymal stem cells originated from mouse ciliary epithelium into retinal neurons by retinol treatment and could be achieved through elevating the expression of the rhodopsin protein and the Nestin, RPE65, and Rhodopsin genes [90]. It was also stated that retinol affects the differentiation potential of gingival progenitor stem cells through activating Wnt/β-catenin signals, and the differentiation of MSCs into neural cells could be achieved by retinol treatment through upregulating the expression of vimentin, Stra13, and RARα/β/γ and downregulating Brachyury expression [91]. To support the present finding, a recent study by Fawzy et al. reported the regenerative potential effect of ascorbic acid retinol using gingival mesenchymal stem/progenitor cells, and their study concluded that the proliferative and chondrogenic differentiation of MSCs by ascorbic acid and retinol was triggered by activating phosphorylated β-Catenin, FOS, EGR1, SGK1, CXCL5, SIPA1L2, TFPI2, KRATP1-5, MT1E, ASNS, and PSAT1 gene expression in gingival mesenchymal stem cells [88]. Comparatively, the cell-loading ability of PVA hydrogel was similar to retinol-loaded PVA hydrogel, which ultimately proved the efficiency of the PVA retinol hydrogel system in the stem cell culture.

## 4. Materials and Methods

### 4.1. Reagents

The hydrolyzed polyvinyl alcohol (PVA), molecular formula [-CH₂CH(OH)-]n and molecular weight 9000–10,000 g/mol (CAS: 9002-89-5) with >98% of hydrolyzation, was acquired from Sigma-Aldrich (Burlington, Vermont, MA, USA). Cosmetic-grade natural pure retinol (or vitamin A) powder, molecular formula C20H30O, CAS:68-26-8, was obtained from NA Beautiful Store, (Shanghai, China).

### 4.2. Aqueous Retinol-Loaded Poly (Vinyl Alcohol) Hydrogels (RPH) Fabrication

**Figure 11:**
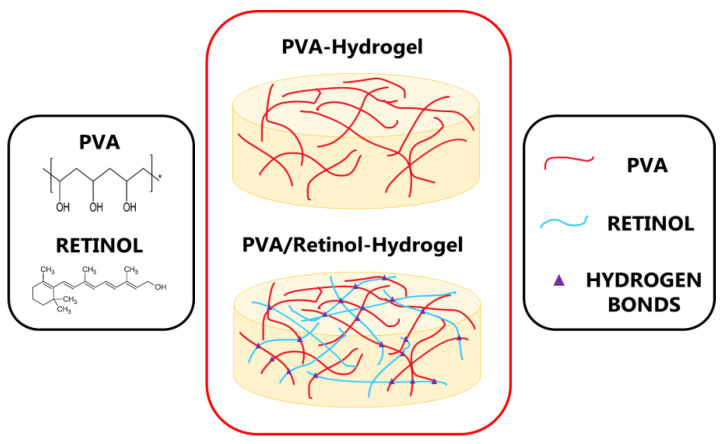
Schematic diagram of the structure of retinol-loaded PVA hydrogels. Purple triangles indicate the possible hydrogen bonding between PVA and retinol molecules.

Hydrogels were fabricated by dissolving the adequate amount of PVA in deionized water (15 wt.%) and stirred for 24 h at 95 °C until a homogenous transparent mixture was obtained. A stock vial containing 200 g of PVA solution at 15 wt.% was fabricated. In total, three different types of retinol-loaded PVA hydrogels (RPH) with varying contents 0.1 wt.% < retinol < 0.5 wt.% were fabricated as follows (Figure 11): Typically, 20, 60, and 100 mg of retinol powder were weighed and then poured into a beaker containing 20 g of PVA solution at 15 wt.% and stirred at room temperature for 30 min until a homogeneous solution was obtained. Samples were labeled from then on as RPHX, where the X indicated the wt.% of retinol of the composite. The sample without retinol was considered as control (PVA solution 15 wt.%).

### 4.3. Characterization

UV-Vis Spectroscopy

The UV-Vis is one of the major experimental techniques employed to date to characterize vitamin-based materials. UV-Vis absorption spectra were recorded on a UV-1800 spectrophotometer (Shimadzu Corporation, Tokyo, Japan) using a quartz cell with an optical path of d ≈ 10 mm. Retinol aqueous solution containing 0.02 wt.% of retinol (0.2 mg/mL), PVA aqueous solution containing 15 wt.% of PVA (150 mg/mL), and PVA/retinol hydrogel containing 150/0.2 mg/mL of PVA and retinol, respectively, were employed for UV absorption. The aqueous solution without sample served as blank control. Spectra were recorded within the wavelength range of 215 nm to 800 nm and systematically background-corrected. All measurements were carried out at room temperature.

### 4.4. In Vitro Retinol Stability and Degradation

Standard in vitro degradation retinol curve was performed as per the procedure reported in the literature [92,93]. First, a calibration standard curve within the 5 mg/L > X > 200 mg/L concentration of retinol in aqueous solution was obtained (Supplementary Figure S1). The curve equation was thus obtained from its linear regression as Y = aX + b, R^2^ > 0.99. From the latter equation, the content of retinol in the solution was calculated using X = (Y − b)/a, where X represents the concentration of retinol in the solution; Y is the corresponding UV absorbance, and the constants of a = 0.0061 and b = 0.0693 are the slope and intercept, respectively. For degradation studies, hydrogels were incubated at 37 °C, and the UV intensity absorption at 334 nm was recorded at different periods. PVA hydrogel without retinol sample served as blank control. The higher retinol concentration (200 mg/L) was chosen based on the maximum UV absorbance (around 1) for the degradation study. 

### 4.5. Textural Properties Analysis

In order to assess the textural properties of the hydrogels, a 25 mL beaker containing 20 g of the RPHX set sample was kept at 4 °C for 1 h before performing textural measurements. Texture profile analyses were performed on a Texture Analyzer by Brookfield Ametek, Model: CT3-50KG with a load cell range of 50 kg (Middleboro, MA, USA). All measurements were carried out using the 12.7 mm cylindrical (AACC std.) TA10 accessory. The operation set-up of the experiment was: 0.5 mm/s compression speed, 5 mm target distance, and 0.1 N trigger force. Subsequently, a single compression cycle was carried out from which the hardness and the consistency of the hydrogel were determined. Indeed, the test measured the force required to obtain a given deformation and correlated these values with their gel structure. When a trigger force of 0.1 N was achieved, the probe began to penetrate to a specified distance (5 mm) after which it returned to its starting position. The maximum force during probe descent provided the material’s hardness (the higher the value, the firmer the sample). Similarly, the area under the curve indicated sample consistency. In this case, the higher the value was related to thicker hydrogel with an associated higher consistency.

### 4.6. Protein Adsorption

The protein adsorption ability of PVA hydrogel was carried out as per our earlier protocol [94]. To estimate the ability of PVA hydrogel in protein adhesion, fetal bone serum (FBS) (100 µL) (Lot No. 2445724RP, Gibco, Carlsbad, CA, USA) was incubated on hydrogel for 2 h at 37 °C. Then, the unbound FBS was gently removed by phosphate-buffered saline (PBS) and washed thrice, and the bound protein was visualized by Coomassie Brilliant Blue G-250 (0.25% CBB G-250 in methanol/water/acetic acid, 40:50:10 volume ratio mixture) for 30 min. The samples without FBS served as negative control. The intensity of CBB G-250 dye was measured by dissolving the stains with a mixture of methanol/water/acetic acid and measured at 590 nm using SpectraMax iD3 (Molecular Devices, LLC., San Jose, CA, USA).

### 4.7. Mineral Deposition

For this experiment, the biomimetic mineral was synthesized by a solid-state reaction from a stoichiometric mixture (Ca/P ratio of 1.72) of calcium carbonate (CaCO_3_, Sigma) (average particle size of <15 µm) and calcium hydrogen phosphate anhydrous (CaHPO4, Sigma). The CaHPO_4_ and CaCO_3_ mixture was heated at 1200 °C (10 °C/minute) for 6 h followed by a cooling rate of 6.5 °C/minute until room temperature. Then, the synthesized biomimetic mineral was powdered to an average particle size of 4.8 µm (Mastersizer APA 2000 E Ver. 5.20, Serial Number: MAL1013724, Malvern Instruments Ltd., Malvern, Worcestershire, UK) [94]. Then, the mineral adhesion of PVA hydrogel was measured using the synthesized biomimetic mineral as follows: The biomimetic mineral was dissolved in DMSO with a concentration of 5 mg/mL and loaded on hydrogel for 24 h. After incubation, the unbound mineral was removed by washing with PBS twice, and alizarin red was used to stain the bound minerals on PVA hydrogel. The amount of mineral deposition was spectrophotometrically quantified after dissolving the mineral stain in a mixture of methanol (20%) and acetic acid (10%) in water for 20 min at 450 nm as per our earlier method [94]. The samples without biomimetic minerals served as negative control. The amount of mineral bound on hydrogel was calculated from the standard curve of synthesized mineral with different concentrations (0.1, 0.5, 1, 2, 3, 4, 5, and 10 mg/mL) (Supplementary Figure S2).

### 4.8. Antimicrobial Activity

The two different bacterial strains, Escherichia coli (*E. coli*) (Gram-negative rod-shaped bacterium) and Staphylococcus aureus (*S. aureus*) (Gram-positive round-shaped bacterium), were used to investigate the antimicrobial activity of PVA hydrogel. The freeze-dried bacterium vial was diluted in the supplement given in the kit (broth and culture plate) and cultured for 24 h, followed by consecutive two cultures using standard broth (10 mL/tubes). Then, the microbial cultures were used for further experiments.

#### 4.8.1. Disk Diffusion Method

The microbial cultures were spread on agar culture plates (60 mm) using a sterile bent rod, and we made a hole in the center of the plate using sterile tips (10 µL) for test samples. The PVA hydrogel was added at two different concentrations (10 and 25 µL), and the antibiotics (20 µL) penicillin and amphotericin B were used as positive controls. After 24 h, the zone of inhibition of PVA hydrogel was measured by using an automatic Interscience Scan 500 zone reader (Model:500, 436000S00871, Interscience International, Saint-Nom-la-Bretèche, Yvelines, Île-de-France, France).

#### 4.8.2. In Vitro Bacterial Attachment Assay

For this, semiquantitative measurement of bacterial adhesion on PVA hydrogel was investigated via a microbial viability assay (MTT) kit. *S. aureus* and *E. coli* were cultured in a nutrient broth medium. For the bacterial attachment assay, PVA hydrogel (200 µL) was added to 24-well culture plates, and bacterial cultures (400 µL) were cultured for 24 h in an incubator. The nonattached bacteria were gently removed by washing with PBS three times. The water-soluble formazan dye by reducing dehydrogenase in bacterial cells was produced by MTT reagent for 2 h, and then the dye was solubilized by DMSO. The amount of dye intensity was measured at 570 nm using SpectraMax iD3 (Molecular Devices, LLC., San Jose, CA, USA)

### 4.9. In Vitro Cell Proliferation

The whole experiment protocol was followed as per the regulatory guidelines of the Institutional Ethics Committee of UCAM-Universidad Católica de Murcia (Authorized No CE051904) UCAM ethics committee (CE nº 052114). All the surgical procedures of bone marrow collection, cell isolation, and phenotype characterization were reported in our previous work [95]. Bone marrow mesenchymal stem (BMMS) cells were isolated from three healthy patients with proper consent, who were scheduled for elective orthopedic surgery. The BMMS cells cultured in DMEM medium supplemented with 1% antibiotics (penicillin and streptomycin) and 10% FBS and incubated in a CO_2_ incubator. For cell proliferation, BMMS cells with a cell density of 5 × 104 were seeded on top of the hydrogels and cultured for 1,4, and 7 days. At the different time points, the cells were treated with MTT reagent (5 mg/mL MTT in PBS); the formazan dye was solubilized with DMSO and measured the OD at 570 nm using SpectraMax iD3.

### 4.10. Cell-Loading Capacity

In order to quantify the maximum number of cells that can load on PVA hydrogel, BMMS cells were loaded on hydrogel by following our previous protocol [94]. The initial cell-loading capacity of hydrogel was measured by loading BMMS cells with a density of 5 × 10^5^ on hydrogel in 24-well culture plates for 6 h. At the end of the experiment, the cells were harvested with 0.25% trypsin and counted by using an automated cell counter (Invitrogen). The percentage of cell loading was calculated from the control group, treating cells without hydrogel.

### 4.11. Fluorescence Microscope

The BMMS cells were seeded on 24-well culture plates and cultured with PVA hydrogel and DMEM medium at different time points 1, 4, and 7 days, respectively. At the end of each time point, the culture medium was removed and washed with PBS three times. Then, the BMMS cells were fixed with 4% paraformaldehyde in PBS for 30 min. After a brief wash with PBS, the BMMS cells’ cytoplasm and nucleus were visualized by fluorophores, such as FITC and DAPI, by standard protocol. The images were captured by using a fluorescence microscope coupled with Axiocam 305 mono (Axio Vert A1, Serial No 3847016567, Carl Zeiss Microscopy GmbH, Suzhou, China)

### 4.12. Statistical Analysis

All the data presented throughout this work were analyzed using one-way analysis of variance (ANOVA) to determine the significant differences between each test (*p* < 0.05). Experiments were carried out at least in triplicate unless otherwise specified, and all results were expressed as a mean ± standard deviation.

## 5. Conclusions

For the first time, the present study investigated the antimicrobial and stem cell proliferative effects of retinol-incorporated PVA hydrogel. The results show that the gel was more stable at 4 °C than at 37 °C, and the degradation rate of retinol was the time-dependent manner with 75 to 78% in 8 days of incubation at 37 °C. In general, the PVA hydrogel with or without retinol showed an inhibitory effect against the tested microbes, whereas the inhibitory effect of PVA hydrogel was more pronounced with the retinol content. The protein and mineral adhesion of PVA hydrogels were improved by 0.5 wt.% retinol, which ultimately supports the suitability of these hydrogels in stem cell cultures. Subsequently, the BMMS cell growth was potentially supported by PVA retinol hydrogel, exhibiting higher BMMS cell proliferation at 0.5 wt.% of retinol-loaded PVA hydrogel. All these data finally show that the fabricated PVA retinol hydrogel could be an excellent biomaterial for culturing stem cells for tissue regeneration applications. Further studies are underway to investigate the osteogenic potential effect of these hydrogels using BMMS cells.

## Figures and Tables

**Table 1:** Gel strength (N) and consistency (mJ) of the retinol-loaded PVA hydrogel.

	Sample	Gel Strength (N)	Consistency (mJ)
At 4 °C	PVA	0.29 ± 0.02	90 ± 20
	RPH0.1	0.25 ± 0.02	50 ± 20
	RPH0.3	0.25 ± 0.02	50 ± 20
	RPH0.5	0.25 ± 0.02	60 ± 20
At 37 °C	PVA	ND	ND
	RPH0.1	ND	ND
	RPH0.3	ND	ND
	RPH0.5	ND	ND

ND—Not detectable. PVA solution 15 wt.%, RPH 0.1-0.1% retinol PVA, RPH 0.3-0.3% retinol PVA, and RPH 0.5-0.5% retinol PVA.

**Table 2:** Antimicrobial activity of retinol-loaded PVA hydrogel by disk diffusion method.

Hydrogels	*E. coli* (mm)	*S. aureus* (mm)
Penicillin 20 µL	14.1 ± 0.27	47.7 ± 1.38
Amphotericin B 20 µL	16.9 ± 0.15	25.0 ± 2.56
PVA 10 µL	-	10.4 ± 0.59
PVA 25 µL	-	12.5 ± 0.24
RPH 0.1 10 µL	8.6 ± 0.22 *	23.1 ± 1.88 *
RPH 0.1 25 µL	10.4 ± 0.27 *	30.4 ± 3.42 *
RPH 0.3 10 µL	9.9 ± 0.16 *	28.2 ± 2.46 *
RPH 0.3 25 µL	15.1 ± 0.31 *	31.0 ± 4.55 *
RPH 0.5 10 µL	10.3 ± 0.48 *	31.3 ± 3.98 *
RPH 0.5 25 µL	14.6 ± 0.39 *	39.3 ± 6.72 *

PVA solution 15 wt.%, RPH 0.1-0.1% retinol PVA, RPH 0.3-0.3% retinol PVA, RPH 0.5-0.5% retinol PVA. * represents statistical significance, * *p* < 0.05 vs. PVA.

## Data Availability

Not applicable.

## Supplementary Materials

The following supporting information can be downloaded at: https://www.mdpi.com/article/10.3390/ijms232415623/s1.
