# Supplementary material for: Retinol-Loaded Poly(vinyl alcohol)-Based Hydrogels as Suitable Biomaterials with Antimicrobial Properties for the Proliferation of Mesenchymal Stem Cells
Source: Int J Mol Sci. 2022 Dec 9;23(24):15623. doi: 10.3390/ijms232415623 (PMC9779207; doi:10.3390/ijms232415623)
Supplement: Supplementary file 1 [file ijms-23-15623-s001.zip › ijms-1908448-supplementary.pdf]

Supplementary Materials

Retinol-Loaded Poly(Vinyl Alcohol)-Based Hydrogels as Suitable Biomaterials with Antimicrobial Properties for the Proliferation of Mesenchymal Stem Cells

Jeevithan Elango [1,2,*], Camilo Zamora-Ledezma [3,*], Daniela Negrete-Bolagay [4], Piedad N. De Aza [5], Vicente M. Gómez-López [3], Ivan López-González [6], Ana Belén Hernández [6], José Eduardo Maté Sánchez De Val [1] and Wenhui Wu [7]

[1] Department of Biomaterials Engineering, Faculty of Health Sciences, UCAM-Universidad Católica San Antonio de Murcia, Campus de los Jerónimos 135, Guadalupe, 30107 Murcia, Spain

[2] Center of Molecular Medicine and Diagnostics (COMManD), Department of Biochemistry, Saveetha Dental College and Hospitals, Saveetha Institute of Medical and Technical Sciences, Saveetha University, Chennai 600077, India

[3] Green and Innovative Technologies for Food, Environment and Bioengineering Research Group (FEnBeT), Faculty of Pharmacy and Nutrition, UCAM-Universidad Católica San Antonio de Murcia, Campus de los Jerónimos 135, Guadalupe, 30107 Murcia, Spain

[4] School of Biological Sciences and Engineering, Yachay Tech University, Urcuquí 100119, Ecuador

[5] Instituto de Bioingeniería, Universidad Miguel Hernández, Avda. de la Universidad s/n, 03202 Elche, Spain

[6] Tissue Regeneration and Repair Group, Biomaterials and Tissue Engineering, Faculty of Health Sciences, UCAM-Universidad Católica San Antonio de Murcia, Campus de los Jerónimos 135, Guadalupe, 30107 Murcia, Spain

[7] Department of Marine Bio-Pharmacology, College of Food Science and Technology, Shanghai Ocean University, Shanghai 201306, China

* Correspondence: srijeevithan@gmail.com or jelango@ucam.edu (J.E.); czamora9@ucam.edu (C.Z.-L.)

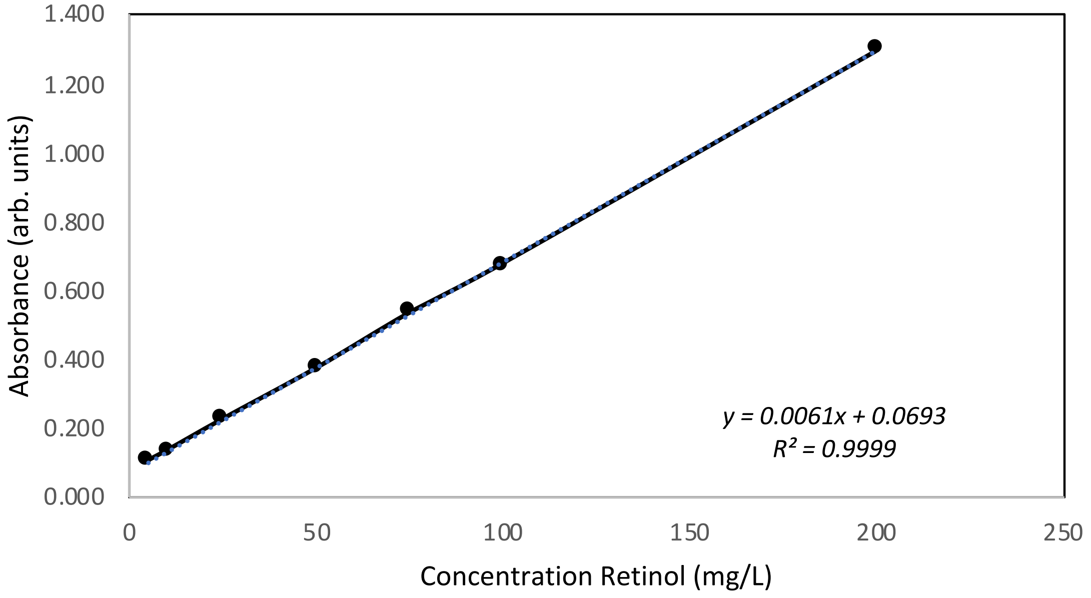

Figure S1. A calibration standard curve within the 5 mg/L > X > 200 mg/L concentration of retinol in aqueous solution at 334 nm

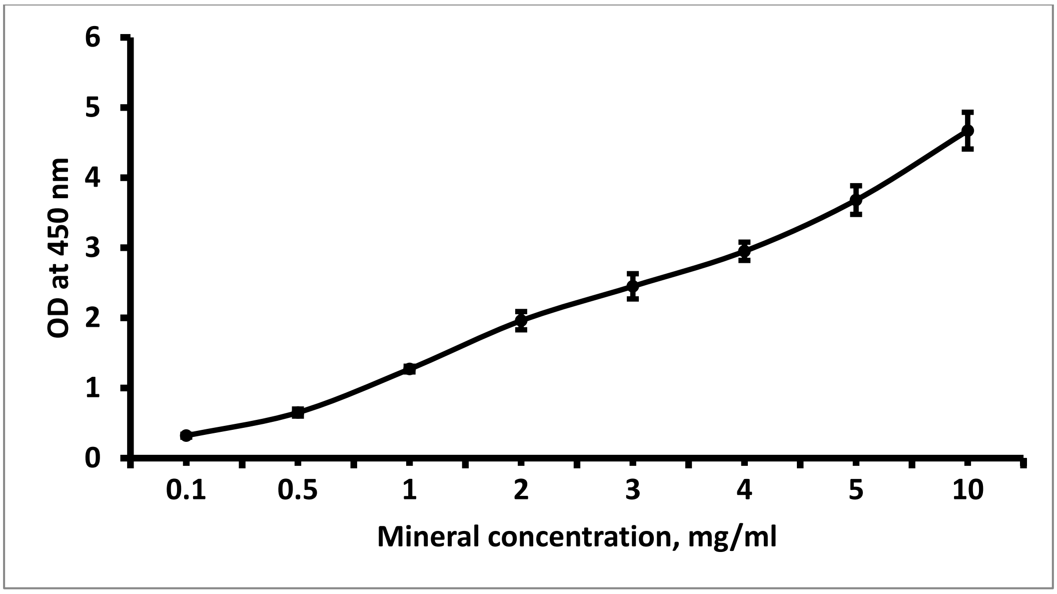

Figure S2. A calibration standard curve of synthesized mineral with different concentration (0.1, 0.5, 1,2,3,4,5 and 10 mg/ml) by alizarin red staining at 450 nm
